# Identification of the long non-coding RNA H19 in plasma as a novel biomarker for diagnosis of gastric cancer

**DOI:** 10.1038/srep11516

**Published:** 2015-06-22

**Authors:** Xiaoying Zhou, Chengqiang Yin, Yini Dang, Feng Ye, Guoxin Zhang

**Affiliations:** 1 Department of Gastroenterology, First Affiliated Hospital of Nanjing Medical University, Nanjing 210029, China; 2 First Clinical Medical College of Nanjing Medical University, Nanjing 210029, China

## Abstract

Recent studies have demonstrated that long non-coding RNAs (lncRNAs) are regarded as useful tools for cancer detection, particularly for the early stage; however, little is known about their diagnostic impact on gastric cancer (GC). We hypothesized that GC-related lncRNAs might release into the circulation during tumor initiation and could be utilized to detect and monitor GC. 8 lncRNAs which previously found to be differently expressed in GC were selected as candidate targets for subsequent circulating lncRNA assay. After validating in 20 pairs of tissues and plasma in training set, H19 was selected for further analysis in another 70 patients and 70 controls. Plasma level of H19 was significantly higher in GC patients compared with normal controls (p < 0.0001). By receiver operating characteristic curve (ROC) analysis, the area under the ROC curve (AUC) was 0.838; p < 0.001; sensitivity, 82.9%; specificity, 72.9%). Furthermore, H19 expression enabled the differentiation of early stage GC from controls with AUC of 0.877; sensitivity, 85.5%; specificity, 80.1%. Besides, plasma levels of H19 were significantly lower in postoperative samples than preoperative samples (p = 0.001). In conclusion, plasma H19 could serve as a potential biomarker for diagnosis of GC, in particular for early tumor screening.

Gastric cancer (GC) is currently the fourth most common malignancy and the second leading cause of cancer death in the world^1^. Few patients could benefit from surgical resection as it is mostly diagnosed at advanced stage and is accompanied by malignant proliferation, extensive invasion and lymphatic metastasis^2^. Therefore, early diagnosis and treatment is an important way to reduce death. Current biomarkers such as serum carbohydrate antigen (CA) 724^3^, and carcinoembryonic antigen (CEA)^4^ are the classic tumor markers commonly used in the management of GC patients. However, these tumor markers have limited utility due to the lack of sufficiently high diagnostic sensitivity and specificity^5^. Therefore, new biomarkers with high sensitivity and specificity in early detection of GC should be explored.

Long non-coding RNAs (lncRNAs), which are longer than 200 bp with no protein-coding capability, play critical roles in tumor initiation, progression and metastasis by modulating oncogenic and tumor-suppressing pathways^6^. Previous studies have proved that lncRNAs are frequently dysregulated expression in different kinds of tumor tissues, including gastric cancer^7^,^8^. Although these lncRNAs have shown great promise as a new kind of tumor markers, they cannot be used for clinical screening purposes because of difficulty in getting biopsies of tissue from GC patients^9^.

Circulating RNA in plasma or serum has been an emerging field for non-invasive diagnostic applications^10^. MiRNAs have been shown to be stable in the blood of cancer patients and regarded as reliable biomarkers in cancer diagnosis^11^. At present, several lncRNAs have also been characterized as potential tumor markers in plasma. For example, lncRNA POU3F3 could serve as a potential biomarker for esophageal squamous cell carcinoma (ESCC) diagnosis, and the combination of POU3F3 and SCCA was more efficient for ESCC detection, in particular for early tumor screening^12^. However, few study investigated circulating lncRNA for early detection of GC patients.

In this study, we selected eight up-regulated and common plasma lncRNA candidates (HOTAIR^13^, CCAT1^14^, PVT1^15^, H19^16^, MALAT1^17^, MRUL^18^, GHET1^19^, HULC^20^) through databases that were previously reported with deregulated expression in gastric cancer. After validation, we found that plasma H19 was upregulated in GC patients. Moreover, we clearly demonstrated that plasma H19 levels are useful to detect GC and monitor tumor dynamics for tumor resection.

## Results

### Study design to detect novel plasma lncRNA biomarker for GC

**Figure 1:**
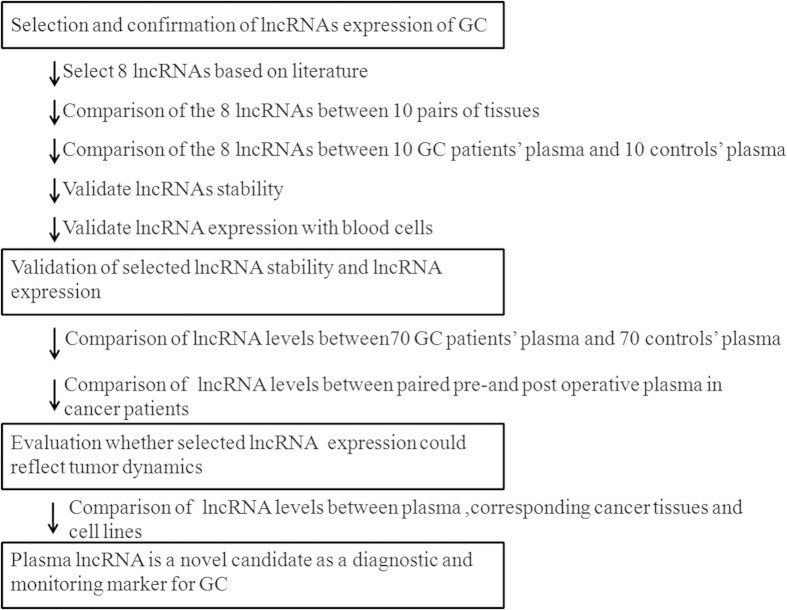
Study design to develop a novel biomarker of plasma lncRNA.

This study was divided into several parts: (1) Selection of 8 lncRNAs from previous published studies in comparisons of GC case and control tissues; (2) Test-scale analyses in tissue and plasma using qRT-PCR in order to validate the expression of selected candidates; (3) Validation of lncRNA stability and correlation with blood cells; (4) large-scale analysis of validating of plasma H19 levels by comparing 70 pairs of GC patients and 26 pairs of dysplasia patients; (5) Evaluation of whether plasma H19 levels reflect tumor dynamics (Fig. 1).

### Selection and detection GC-related lncRNAs

**Figure 2:**
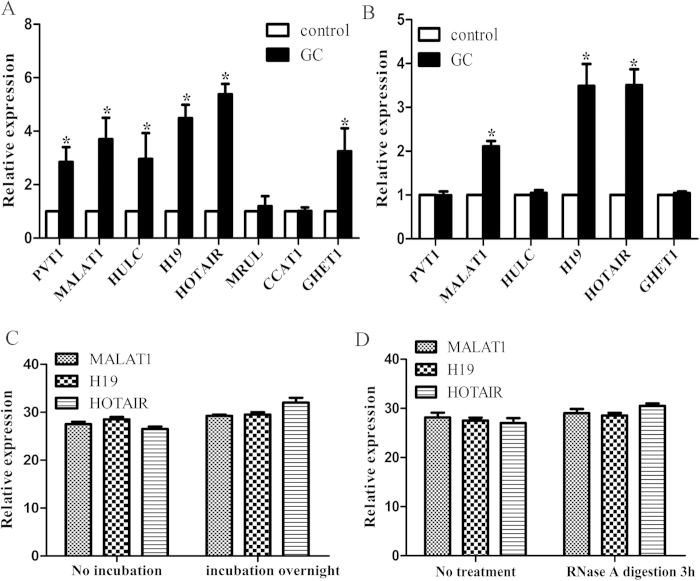
Validation of GC-related lncRNAs expression as circulating biomarkers. (**A**) The expression of lncRNAs in GC tissues. Differential expressions of lncRNAs in GC tissues were compared with those of normal tissues. (**B**) The expression of lncRNAs in GC plasma. Differential expressions of lncRNAs in GC plasma were compared with those of normal plasma. (**C**) LncRNAs expression with prolonged room temperature incubation time. (**D**) LncRNAs expression with RNase A digestion. (*P < 0.05, compared with control tissues).

On the basis of previous study, 8 lncRNAs (HOTAIR, CCAT1, PVT1, H19, MALAT1, MRUL, GHET1, HULC) which have been reported to be differently expressed in GC were selected in this study. To define the dynamic range and sensitivity of lncRNA quantification by real-time PCR, the synthetic lncRNA probes were serially diluted 10-fold from concentrations of 0.1 to 0.000001 fmol for the 8 lncRNAs. The linearity of the quantitative RT-PCR between the logarithmic values of the lncRNAs and the Ct values was confirmed for each lncRNA (R^2^ > 0.99 for each lncRNA, Supplementary Figure 2). All the 8 lncRNAs were then subjected to qPCR validation using 20 pairs of GC and control tissues. Among them, HOTAIR, PVT1, H19, MALAT1, GHET1 and HULC were significantly higher in tumor tissues compared with control tissues. However, CCAT1 and MRUL did not show any significant different expression between the two groups and therefore were eliminated in the subsequent study (Fig. 2A). Next, we investigated the 6 lncRNAs expression in 20 pairs of GC and control plasma. Among them, MALAT1, H19 and HOTAIR were significantly higher in plasma of tumor patients, while the other 3 lncRNAs showed no significant changes (Fig. 2B). We then determined the stability of the 3 lncRNAs in the plasma. Plasma samples were left under conditions including incubation at room temperature for 0, 6, and 24 h or with RNase A for 3 h to determine whether lncRNAs could be degraded. Results indicated that longer incubation time (Fig. 2C) or RNase A treatment (Fig. 2D) had hardly any effect on plasma level of HOTAIR and H19, but MALAT1 expression decreased slightly, which means that MALAT1 might not be stable in the plasma and thus it was ruled out for our future study.

**Figure 3:**
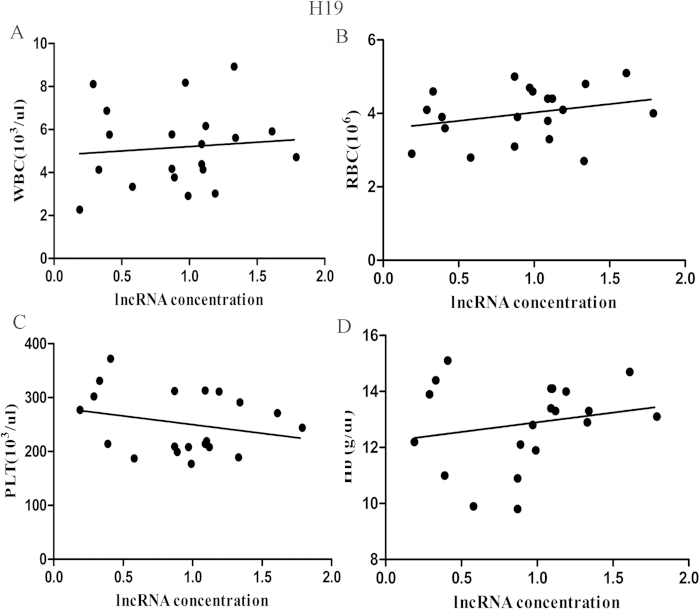
Correlation between plasma H19 concentrations and the haematocytes of peripheral blood in GC patients. There was no significant correlation between plasma H19 concentrations and any type of peripheral haematocytes and plasma (P > 0.05).

Recent reports demonstrated that some circulating ncRNAs may be derived from peripheral blood cells. We evaluated the correlation between the plasma levels of the lncRNAs and peripheral blood cells in 20 GC patients. We found a significant correlation between the plasma HOTAIR levels and concentrations of white blood cells and platelets in the peripheral blood (Supplementary Figure 3) and no significant correlation was observed between plasma H19 levels and any type of blood cells (Fig. 3). Considering the influence of secretion of lncRNAs from blood cells or haemolysis on the results of clinical application, we chose H19 for our further analyses.

### Large-scale analysis of validation of plasma H19 levels by comparing GC patients with healthy controls

**Figure 4:**
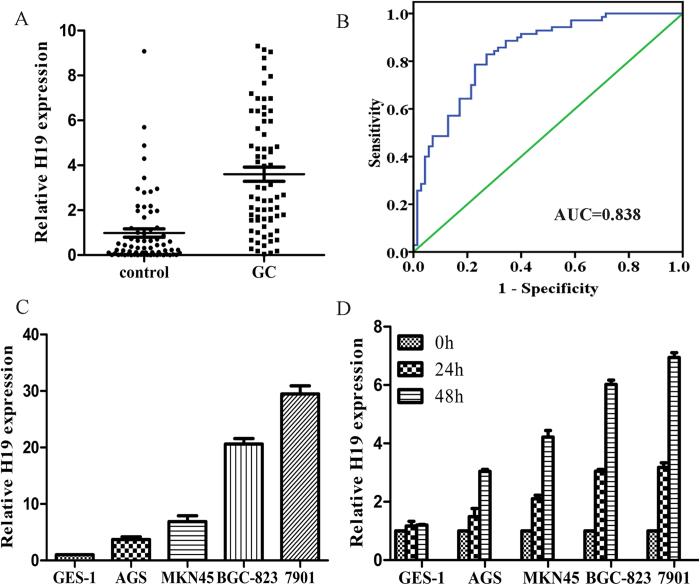
Evaluation of H19 levels in GC cell lines and plasma samples of patients with GC, GC cell lines or culture medium. (**A**) Plasma H19 levels in 70 consecutive GC patients and 70 healthy controls. Using a real-time RT-PCR assay, difference in plasma H19 expression between GC patients and normal healthy controls was determined (P < 0.0001). (**B**) The ROC curve analysis for discriminative ability between GC cases and normal controls (AUC = 0.838, sensitivity: 0.829, specificity: 0.729). (**C**) The expression of H19 in GC cell lines. Differential expressions of H19 in GC cell lines were compared with GES-1 cells (P < 0.05, compared with GES-1). (**D**) The expression of H19 in GC cell culture medium incubated for 0, 1, 2 days (P < 0.05, compared with GES-1).

Next, plasma H19 levels were examined on a large scale for our validation study using plasma from 70 GC patients and 70 healthy controls by qRT-PCR assays. Result showed that H19 expression was significantly higher in GC patients than that in controls (Fig. 4A, P < 0.0001). Representation of the data using an ROC plot showed strong separation between the two groups, with an AUC of 0.838 (0.772–0.903). The sensitivity and specificity was 0.829 and 0.729, respectively (Fig. 4B). We also examined the association of plasma H19 concentrations with clinicopathological factors in all 90 GC patients. Table 1 shows expression levels of H19 in plasma. However, we did not find any significant correlations between the H19 level and clinical factors.

To further test the hypothesis that plasma lncRNAs were primarily released or leaked from the tumor cells, qPCR analysis was used to measure H19 expression in different five GC cell lines (including four GC cells: 7901, AGS, BGC-823, MKN45, and one normal human gastric epithelial cell line: GES-1), and each cell culture medium which was incubated for 0, 24 h and 48 h. We found that H19 expression was significantly higher in GC cells compared with GES-1 (Fig. 4C). H19 in culture medium was also increased with the incubation time in GC cells, but not in GES-1 cell (Fig. 4D).

### Further study on clinical application of plasma H19 in early stage GC patients undergoing endoscopic resection

**Figure 5:**
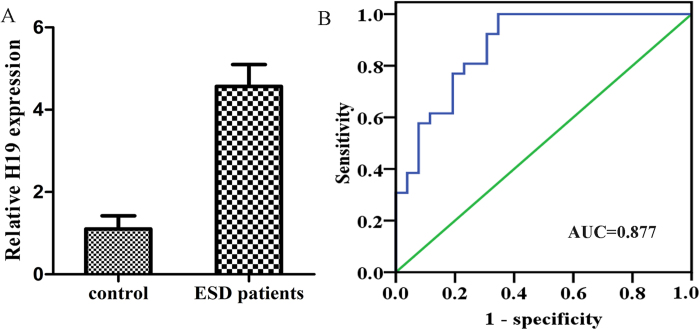
(**A**) Plasma H19 expression level increased in early stage GC patients (P < 0.0001). (**B**) The ROC curve analysis for discriminative ability between early stage GC cases and normal controls (AUC = 0.877, sensitivity: 0.855, specificity: 0.801).

To further evaluate the possibility of clinical application of plasma H19, H19 levels in 26 dysplasia patients who underwent endoscopic submucosal dissection (ESD) and 26 healthy controls were examined by qPCR. The plasma H19 levels were significantly higher in dysplasia patients (p = 0.000) (Fig. 5A). The value for the AUC for the plasma H19 analysis was 0.877, with a sensitivity of 85.5% and specificity of 80.1% (Fig. 5B). Indeed, the sensitivity of plasma H19 for dysplasia detection was much higher than that of conventional tumor markers, including CEA and CA199 at our institute (data not shown). Our results provided evidence that plasma H19 levels can be used to distinguish early stage GC patients from controls to a clinically satisfactory degree compared with conventional tumor markers.

### Evaluation of the use of H19 for monitoring tumor dynamics in GC patients

**Figure 6:**
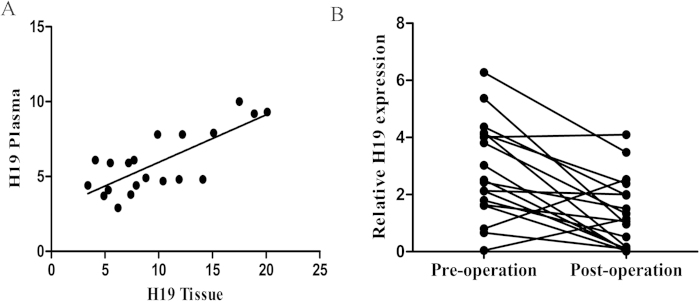
(**A**) Spearman’s rank correlation scatter plot of GC-related lncRNAs levels in tumor samples and plasma (r^2^ = 0.587, P < 0.0001). (**B**) Comparison of plasma H19 concentrations between pre- and postoperative samples from GC patients (p = 0.001).

To clarify the correlation of H19 expression between plasma and GC tissue, qPCR analysis was used to measure the GC-related lncRNAs expression in 20 GC tumor tissues and paired plasma samples, and then correlation between the two groups of H19 level was analyzed. A moderate significant correlation was observed for H19 (r^2^ = 0.587, p < 0.0001), which was consistent with our previous hypothesis (Fig. 6A).

Moreover, the expression level of H19 was analyzed in 18 paired pre- and postoperative plasma samples from GC patients who underwent gastrectomy and its expression was found to be significantly reduced in postoperative samples (p = 0.001) (Fig. 6B). These findings indicated that the level of H19 in plasma might reflect the expression in the tumor.

## Discussion

Recently, it has been demonstrated that the nucleic acids are detectable in plasma of cancer patients and therefore may be utilized as a tool for cancer diagnosis^21^. Numerous studies have focused on miRNAs as potential tumor markers for cancer diagnosis and prognosis prediction^22^,^23^. However, little is known about lncRNAs diagnostic utility in plasma.

Circulating lncRNAs were thought to be unstable because of the high level of RNase activity in plasma, and in cancer patients, increased plasma RNase has been detected^24^. In the present study, we confirmed that circulating lncRNAs were remarkably stable even when treated directly with RNase A digestion. These findings were consistent with those in patients with prostate cancer and ESCC. However, the precise mechanism used to explain why circulating lncRNAs are resistant to endogenous RNase digestion remains largely unknown. One explanation is that they are packaged in some kinds of microparticles, such as exosomes, microvesicles, apoptotic bodies, and apoptotic microparticles^25^. Recently, Pritchard CC reported the caution in a cancer biomarker study of circulating ncRNAs because circulating ncRNAs may be derived from peripheral blood cells^26^. Therefore, we evaluated the correlation between plasma H19 concentrations and haematocytes of peripheral blood in consecutive GC patients. As a result, there was no significant association between plasma H19 concentrations and any types of peripheral haematocyte. This result indicated that H19 expression in plasma may reflect tumor dynamics in GC patients.

To date, circulating lncRNA has been investigated in several cancers as diagnostic marker, such as ESCC^12^ and prostate cancer^27^. However, only two studies have examined gastric lncRNAs in patients with GC and sought to identify lncRNAs that are predictive for the diagnosis or prognosis of GC^28^,^29^. Expression levels of lncRNA AA174084 were down-regulated significantly in 95 of 134 GC tissues (71%) compared with the levels in paired, adjacent, normal tissues and it may have potential as marker for the early diagnosis of GC^28^. Another study suggested that lncRNA H19 expression was significantly higher in GC patients and reduced in postoperative samples^29^. In the present study, we recruited more GC patients, which made the results more convincing. Besides, we also included 26 early stage GC patients identified by ESD procedures. Our results provided further evidence that plasma H19 levels can be used to distinguish early stage GC patients from controls to a clinically satisfactory degree compared with conventional tumor markers.

In the present study, the initial GC-related lncRNAs screening was performed based on different expression profiling between GC tumor samples and matched normal samples that have been demonstrated in previous studies. The selected lncRNAs were subjected to qPCR validation. The 8 lncRNAs were identified and then further measured their expression levels in plasma and tissue from GC patients and healthy subjects. The results demonstrated that the levels of H19, HOTAIR and MALAT1 were significantly higher in plasma from GC patients compared with normal controls, providing strong evidence that GC-related lncRNAs could be released into the circulation and that their different expression profiles in plasma could be used as diagnostic markers for GC. Among the three lncRNAs, H19 showed more stable in the blood and no significant correlation with any type of blood cell in the peripheral blood. Consequently, we chose H19 for our further study. We also detected miR-675 expression in plasma. H19 is the precursor of miR-675 in GC. We found that miR-675 expression was also higher in GC plasma. However, after we incubated plasma samples at room temperature for 0, 6, and 24 h, miR-675 expression decreased significantly (data not shown), which means that miR-675 might not be stable in the plasma and thus it was ruled out for our future study. It is possible that the efficiency with which H19 is processed into miRNAs may vary significantly during different developmental stages and/or in different tissues^30^. H19 provided the high diagnostic power for detection of GC (AUC = 0.838; sensitivity, 82.9%; and specificity, 72.9%), suggesting that plasma H19 could serve as a promising tumor marker for GC detection. Furthermore, we found that H19 could well distinguish early stage tumor, with a much higher sensitivity and specificity than conventional biomarkers. This is the first time to systematically characterize circulating lncRNAs in plasma as diagnostic markers for GC.

Next, we investigated whether plasma H19 concentrations could reflect tumor dynamics in GC by two different analyses. One is the comparison between expression of H19 in plasma and primary tumor tissue, which demonstrated that plasma and primary GC tissue samples showed that high levels of plasma H19 represented higher expressions in primary GC tissues than in normal mucosa. Second analysis involves comparison of plasma H19 concentrations in paired plasma obtained before and after surgery. As a result, concentrations of H19 were significantly reduced postoperatively in patients with high preoperative plasma H19. These findings clearly demonstrated that plasma concentrations of H19 reflect tumor dynamics and are available as a new plasma biomarker for monitoring tumor status. Longer period of the patients should be observed and detect lncRNA expression if there was recurrence.

Taken together, we clearly demonstrated that plasma levels of H19 may potentially be useful for cancer screening and monitoring tumor dynamics in GC patients postoperatively. Nevertheless, many issues must be addressed before these findings can be translated into a clinically useful, non-invasive screening strategy for GC patients. We will prospectively confirm the usefulness of plasma H19 in a large number of patients and report on these results in the near future. Furthermore, if possible, more sensitive biomarkers in plasma lncRNAs should be detected for translation into the clinical setting of GC.

## Methods

### Ethics Statement

All the experiments were performed in accordance with relevant guidelines and regulations. Written informed consent was obtained from each participant prior to blood and tumor samples collection. All of the clinical samples were obtained from First Affiliated Hospital of Nanjing Medical University. This study was approved by the Ethical Review Committee of Nanjing Medical University.

### Patients and samples

In this study, consecutive hospitalized patients who had newly diagnosed with GC were selected from First Affiliated Hospital of Nanjing Medical University between January 2013 and September 2014. All patients selected met the following inclusion criteria: pathological examination confirmed primary GC by available biopsy samples; and no chemotherapy treatments were given before surgery. Patient characteristics with respect to age, sex, smoking status, drinking status, TNM stages and metastasis are described in Table 1. Preoperative plasma samples were collected from GC patients. All patients were pathologically diagnosed as having GC using surgical specimens and biopsies. As a control, plasma was collected from 90 healthy participants. They underwent medical examinations and did not have any other cancerous disease. The stage of tumors was assessed according to the Union of International Control of Cancer (UICC) classification.

Fresh tumor tissues and paired adjacent normal tissues were obtained from GC patients and were immediately frozen in liquid nitrogen and then stored at −80 °C until RNA extraction. Peripheral blood (7 ml) was obtained from each patient at the time before the gastrectomy or endoscopic resection and from the healthy controls. Blood was collected from patients and controls in sodium heparin tubes (BD Vacutainer, Becton, Dickinson and Company, Franklin Lakes, NJ, USA) and immediately subjected to the three-spin protocol (1500 rpm for 30 min, 3000 rpm for 5 min, and 4500 rpm for 5 min) to prevent contamination by cellular nucleic acids.

### RNA extraction and quantitative real-time PCR (qPCR)

The plasma was separated from venous blood within 12 hours. Three cell lines were cultured and a fraction of the culture media was collected at 0, 1 and 2 day after the initial seeding of cells in 10 cm dishes. The plasma and culture media samples were further resolved by a 15-min centrifugation at 12000 rpm at 4 °C to completely remove cell debris. Following the manufacturer’s protocol, RNA was extracted using Trizol Reagent (TaKaRa, Japan). The reverse transcription reaction was carried out using PrimeScript II cDNA synthesis kit (Takara, Japan) in 15 μl solution containing 5 μl of RNA extract. For cDNA synthesis, the reaction mixtures were incubated at 16 °C for 30 min, at 42 °C for 30 min, and at 85 °C for 5 min and then held at 4 °C. The quantitative detection of lncRNA was performed using SYBR as implemented in the ABI Step one Real-Time PCR System (Applied Biosystems), and the reaction mixtures were incubated at 95 °C for 10  min, followed by 40 cycles of 95 °C for 15 s, and 60 °C for 1 min. The primers of lncRNAs were listed in supplementary table 1. The plasma lncRNA concentrations were calculated using a standard curve constructed using lncRNA probes. The expression of lncRNAs from tissue samples was normalized using the 2^−ΔΔCT^ method relative to GAPDH. ΔCt was calculated by subtracting Ct values of GAPDH from the lncRNAs. ΔΔCt was then calculated by subtracting ΔCt of normal tissue from ΔCt of GC tissues. The change in gene expression was calculated using the equation 2^−ΔΔCt^.

### Cell culture

GC cell lines, AGS, MKN45, 7901 and BGC-823 and human gastric immortalized epithelial GES-1, were purchased from Shanghai Cell Institutes (Shanghai, China) and were authenticated by STR analysis to prevent contamination (Supplementary Figure 1). They were cultured in RPMI medium 1640 (Invitrogen, Carlsbad, CA) containing 10% fetal bovine serum (Gibco, Grand Island, NY) and 1% penicillin-streptomycin at 37 °C in 5% CO_2_. Cells were plated in 6-well plate at a density of 2 × 10^5^ per well, and then the medium was switched to fresh RPMI-1640 12  h after plating. After incubation for 3 days, the cells and the cell culture media were separately collected for RNA isolation.

### Statistical analysis

All statistical analyses were performed using SPSS 18.0 (SPSS, Chicago, Illinois, USA). The Mann-Whitney test was used to compare differences in plasma lncRNA concentrations between the cancer group and healthy group, and the Wilcoxon test was used to compare the paired plasma samples before and 14 days after gastrectomy. A P-value of 0.05 was considered significant. Receiver-operating characteristic (ROC) curves and area under the curve (AUC) were used to assess the feasibility of using plasma lncRNA concentrations as a diagnostic tool for detecting GC.

## Additional Information

**How to cite this article**: Zhou, X. *et al.* Identification of the long non-coding RNA H19 in plasma as a novel biomarker for diagnosis of gastric cancer. *Sci. Rep.* **5**, 11516; doi: 10.1038/srep11516 (2015).

## Supplementary Material

Supplementary Information

## Figures and Tables

**Table 1:** GC patients’ characteristics and plasma H19 concentrations.

**Variables**	**Patients (n=90)**	**Plasma H19 (mean)**	**P value**
**Ages (years)**			
<60	44	3.872	0.816
≥60	46	3.699	
**Sex**			
Male	57	3.673	0.351
Female	33	4.003	
**Smoking**			
Yes	41	3.791	0.911
No	49	3.662	
**Drinking**			
Yes	36	3.883	0.881
No	54	3.619	
**TNM stage**			
I-II	33	3.564	0.412
III-IV	57	3.986	
**Metastasis**			
M_0_	40	3.415	0.198
M_1_	50	4.066	
