# Supplementary material for: Identification of the long non-coding RNA H19 in plasma as a novel biomarker for diagnosis of gastric cancer
Source: Sci Rep. 2015 Jun 22;5:11516. doi: 10.1038/srep11516 (PMC4476094; doi:10.1038/srep11516)

**Identification of the long non-coding RNA H19 in plasma as a novel biomarker for diagnosis of gastric cancer**

Xiaoying Zhou^1, 2^*; Chengqiang Yin^1, 2^*; Feng Ye^1, 2^*; Yini Dang^1, 2^; Guoxin Zhang^1§^

Supplementary Table 1. Sequences of primers of the eight lncRNAs

| Name | Sequences | |
| --- | --- | --- |
| qPCR primers | | |
| H19 | Sense | 5'- ATCGGTGCCTCAGCGTTCGG-3' |
|  | Anti-sense | 5'- CTGTCCTCGCCGTCACACCG-3' |
| CCAT1 | Sense | 5’-TTTATGCTTGAGCCTTGA-3’ |
|  | Anti-sense | 5’-CTTGCCTGAAATACTTGC-3’ |
| HOTAIR | Sense | 5'- CAGTGGGGAACTCTGACTCG-3' |
|  | Anti-sense | 5'- GTGCCTGGTGCTCTCTTACC-3' |
| MALAT1 | Sense | 5'- CAGTGGGGAACTCTGACTCG -3' |
|  | Anti-sense | 5'- GTGCCTGGTGCTCTCTTACC-3' |
| PVT1 | Sense | 5’-CATCCGGCGCTCAGCT-3’ |
|  | Anti-sense | 5’-TCATGATGGCTGTATGTGCCA-3’ |
| MRUL | Sense | 5’-ACCCACAGACAACTGTGGACCC-3’ |
|  | Anti-sense | 5’-GCCGCCCCTATTGTTGCCCA-3’ |
| GHET1 | Sense | 5’-CCCCACAAATGAAGACACT-3’ |
|  | Anti-sense | 5’-TTCCCAACACCCTATAAGAT-3’ |
| HULC | Sense | 5’- ATCTGCAAGCCAGGAAGAGTC-3’ |
|  | Anti-sense | 5’- CTTGCTTGATGCTTTGGTCTGT-3’ |
| GAPDH | Sense | 5'- TGTGTTGGCGTACAGGTCTTTG-3' |
|  | Anti-sense | 5'- GGGAAATCGTGCGTGACATTAAG -3' |

Supplementary Figure 1. GC cell lines authentication by STR analysis. The results showed no cross-contamination of other human cell line is found and 100% matched cell lines are found in ATCC, DSMZ and JCRB data banks.


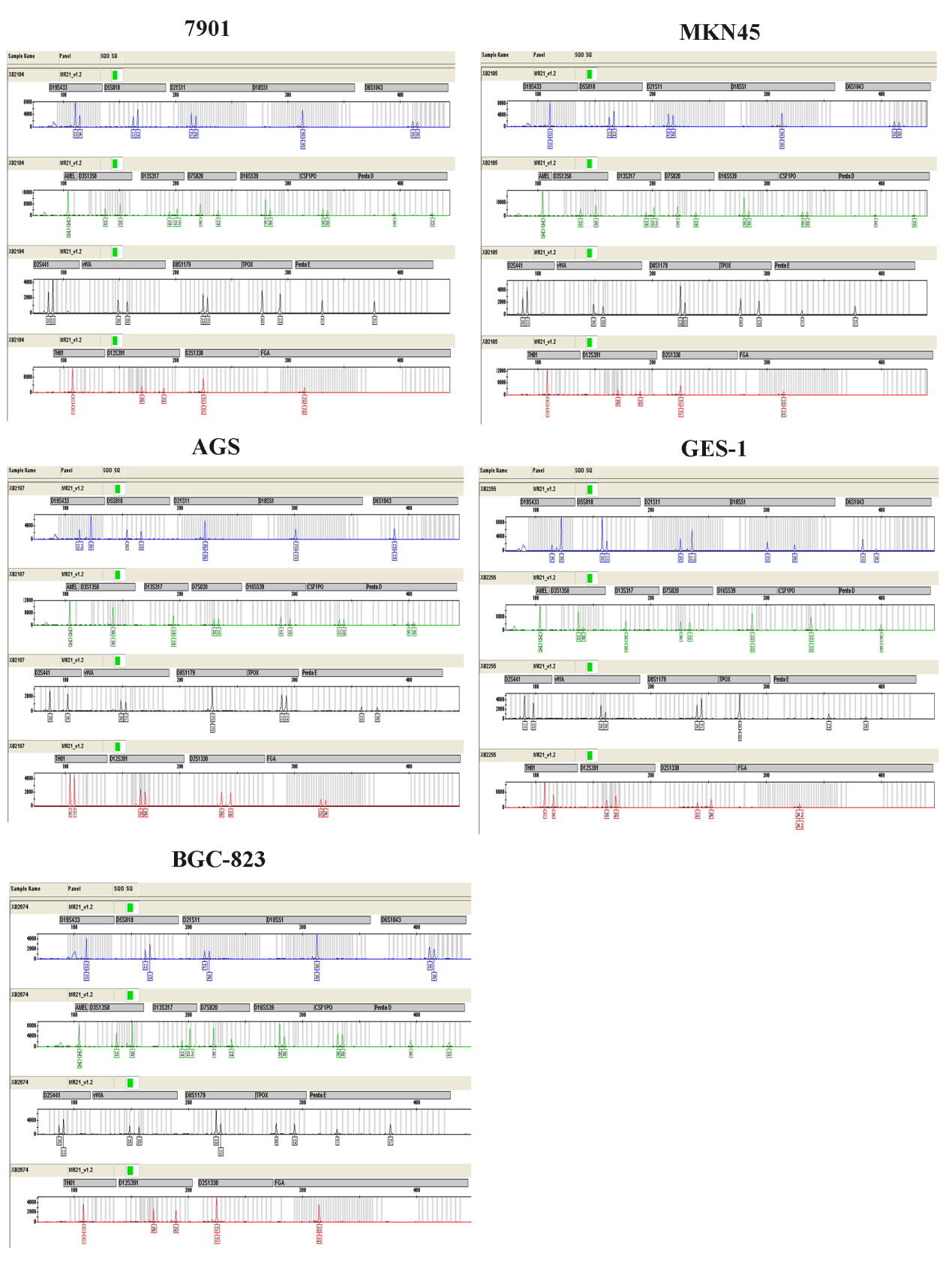


Supplementary Figure 2. Standard curve of the 8 lncRNAs using synthetic lncRNA probes. Ten-fold serial dilution of synthetic lncRNA probes was used to generate the standard curves. Linearity was confirmed within these concentrations for the 8 lncRNAs ranging from 0.1 to 0.000001 fmol. (H19: y=-3.100x+18.00 (R^2^=0.9966); PVT1: y=-3.229x+17.86 (R^2^=0.9848); MALAT1: y=-2.962x+17.52 (R^2^=0.9923); HULC: y=-3.100x+18.83 (R^2^=0.9939); HOTAIR: y=-2.903x+18.98 (R^2^=0.9909); MRUL: y=-2.971x+19.44 (R^2^=0.9841); CCAT1: y=-3.314x+18.71 (R^2^=0.9909); GHET1: y=-3.157x+19.19 (R^2^=0.9899)).


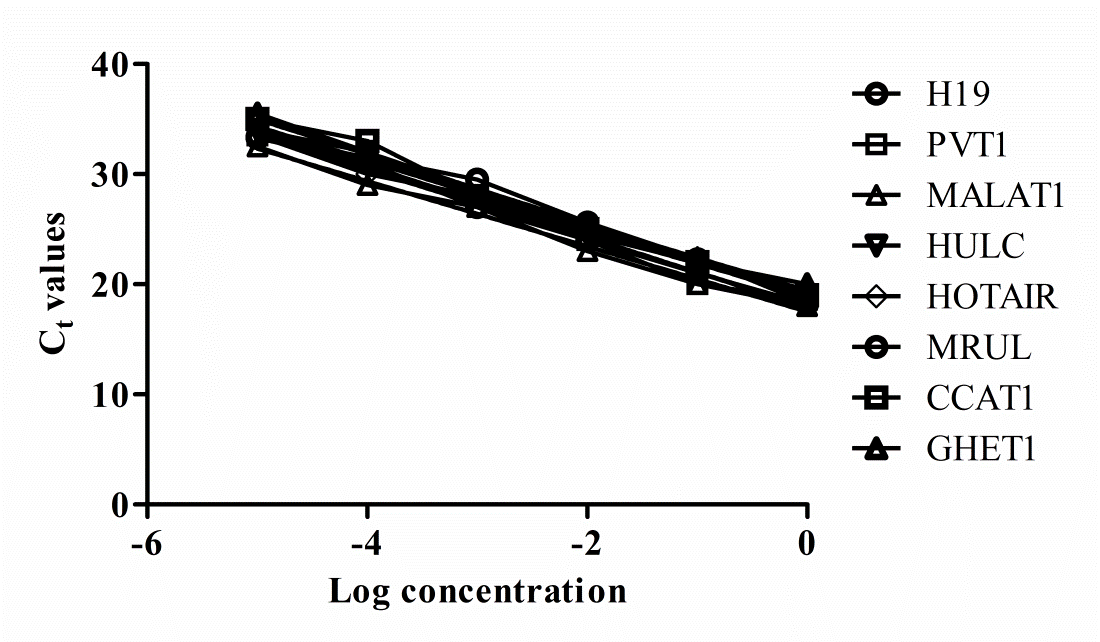


Supplementary Figure 3. Correlation between plasma HOTAIR concentrations and the haematocytes of peripheral blood in GC patients. A significant correlation was observed between plasma HOTAIR levels and concentrations of white blood cells (P=0.038) and platelets (P=0.0303) in the peripheral blood.


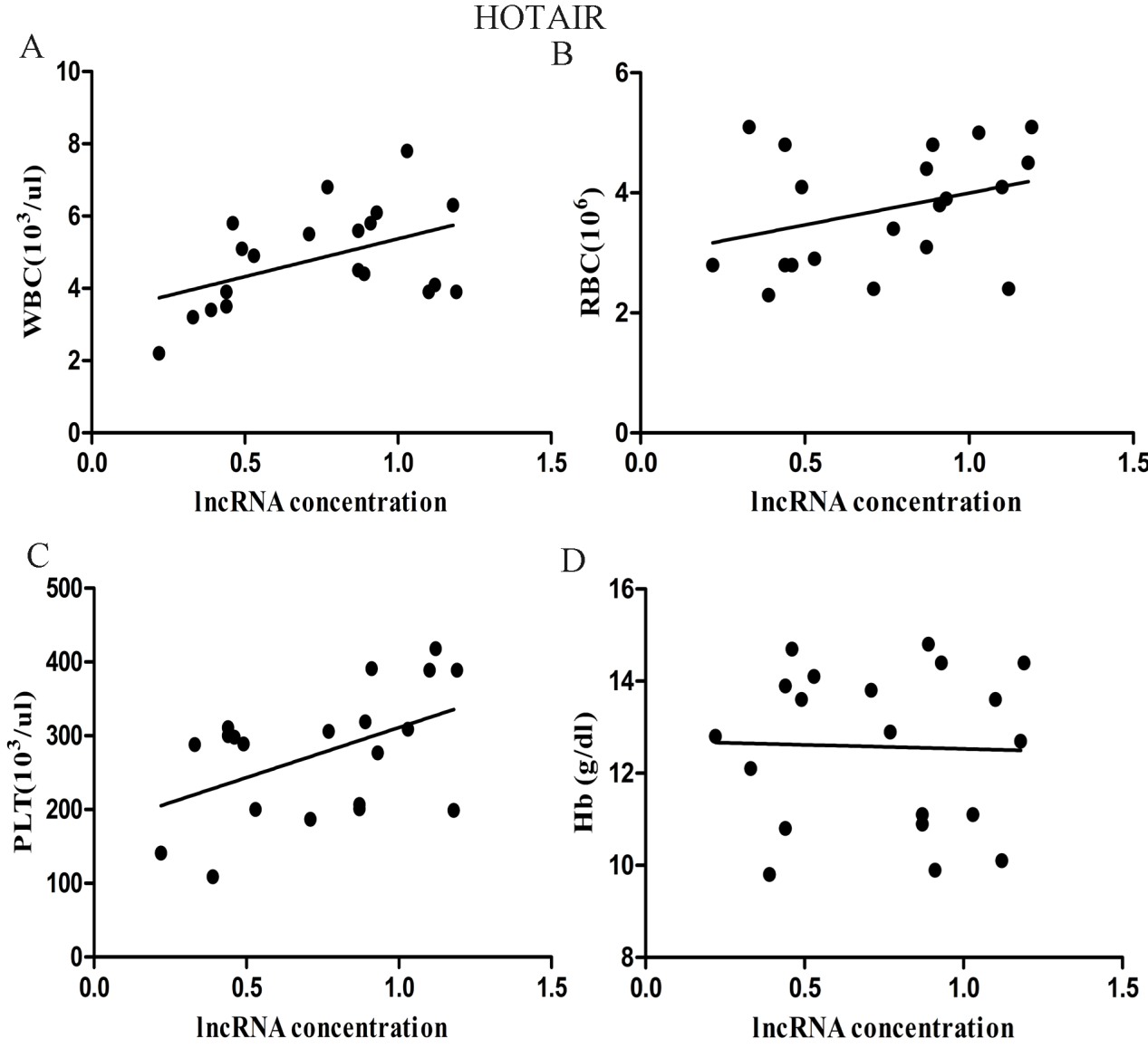

Supplement: Supplementary Information [file srep11516-s1.doc]
